# Idiosyncratic Drug-Induced Liver Injury (IDILI): Potential Mechanisms and Predictive Assays

**DOI:** 10.1155/2017/9176937

**Published:** 2017-01-04

**Authors:** Alexander D. Roth, Moo-Yeal Lee

**Affiliations:** Department of Chemical & Biomedical Engineering, Cleveland State University, 1960 East 24th Street, Cleveland, OH 44115-2214, USA

## Abstract

Idiosyncratic drug-induced liver injury (IDILI) is a significant source of drug recall and acute liver failure (ALF) in the United States. While current drug development processes emphasize general toxicity and drug metabolizing enzyme- (DME-) mediated toxicity, it has been challenging to develop comprehensive models for assessing complete idiosyncratic potential. In this review, we describe the enzymes and proteins that contain polymorphisms believed to contribute to IDILI, including ones that affect phase I and phase II metabolism, antioxidant enzymes, drug transporters, inflammation, and human leukocyte antigen (HLA). We then describe the various assays that have been developed to detect individual reactions focusing on each of the mechanisms described in the background. Finally, we examine current trends in developing comprehensive models for examining these mechanisms. There is an urgent need to develop a panel of multiparametric assays for diagnosing individual toxicity potential.

## 1. Introduction

Adverse drug reactions (ADRs) are among the five leading causes of death in the United States, with hepatotoxic events being the most common site of ADRs, owing to the fact that the liver is the organ associated with clearance of toxic substances [1]. While the majority of ADRs that contribute to acute liver failure (ALF) are considered to be dose-dependent or “intrinsic,” roughly 10–15% of ALF can be attributed to individual effects that are not dependent on dose [1]. These idiosyncratic adverse drug reactions (IADRs) are responsible for a significant amount of drug withdrawals during and after postclinical marketing trials [1]. Additionally, the cost for successful drug development can range from $160 million to $1.8 billion, and it takes ten to fifteen years from lead compound discovery to clinical evaluation [2]. This cost increases significantly when drug candidates fail at the late stage of clinical trials or drugs are withdrawn from the market due to unexpected ADRs. With roughly a postmarketing failure rate of one drug per year, there is a critical need to reduce the human and fiscal cost by decreasing incidence of IADRs.

While significant resources have been put into developing toxicity screens, there is very little in the way of predicting IADRs [3]. Many of the current technologies for detecting hepatotoxicity focus on cytotoxicity screens for in vitro hepatocyte cultures as a method to weed out drug candidates before clinical trials [3]. Subsequent follow-up with animal models is used to reduce the possible drug failure [4–6]. Unfortunately, these screens often have poor predictive value in assessing hepatotoxicity potential [7]. This problem becomes exacerbated when accounting for IADRs, as many of the preclinical trials focus on models that utilize healthy livers with fully functioning drug metabolizing enzymes (DMEs) [8–10].

It is believed that propensities for IADRs and IDILI can be increased by both genetic and nongenetic factors. Such nongenetic factors could include current disease states, pregnancy, other drugs being simultaneously taken with the drug causing the adverse reaction, and age [11]. Potential genetic factors focus on polymorphisms affecting the various DMEs, enzymes that reduce reactive oxygen species (ROS), drug transporters, the inflammation response in the liver, and the major histocompatibility complex (MHC) class of proteins [5, 11]. Aside from several of the mechanisms that govern immune responses, all of these genetic responses can be localized to the liver, although a few other mechanisms can be found in other organs.

In this review, we address the basis for drug metabolism and disposition in the liver and the proteins and enzymes involved in these processes. We discuss any polymorphisms that have been correlated with (and potentially causative of) ADRs and assays that detect potential for ADRs due to these polymorphisms. The potential mechanisms addressed will include mutations that affect drug metabolism, drug disposition, antioxidant mediating enzymes, and the immune system. In addition, we will address what demographics are most likely to contain these mutations. Next, we discuss the various assays that can be implemented for measuring responses associated with dysfunction in critical proteins. Finally, we discuss the design of future platforms that can help integrate these assays to generate a complete profile for predicting ADRs.

## 2. Mechanisms for Idiosyncratic Hepatotoxicity

**Figure 1:**
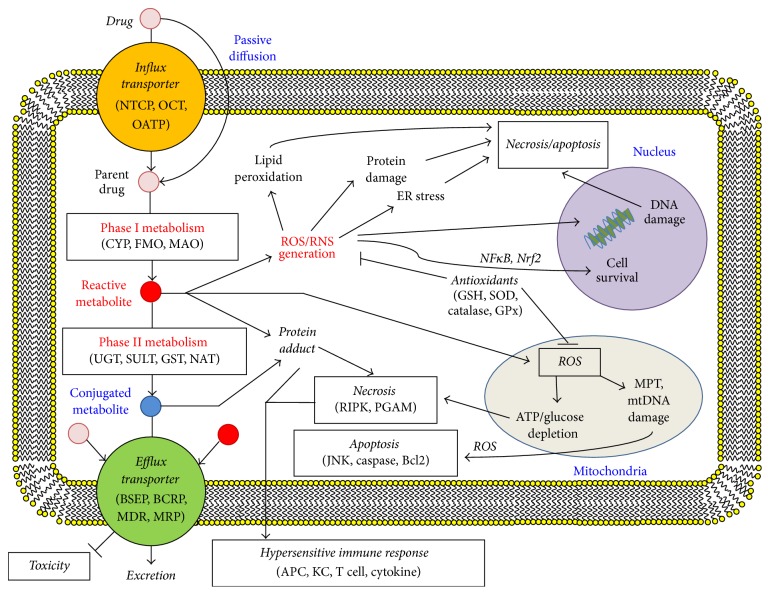
Simplified mechanisms of drug metabolism in liver cells with potential pathways towards toxicity. Abbreviations are used as follows: Na^+^-taurocholate cotransporting polypeptide (NTCP), organic cation transporter (OCT), organic anion transporting polypeptide (OATP), bile salt export pump (BSEP), breast cancer resistance protein (BCRP), multidrug resistance protein (MDR), multidrug resistance-associated protein (MRP), cytochrome P450 (CYP), flavin-containing monooxygenase (FMO), monoamine oxidase (MAO), UDP-glucuronosyltransferases (UGT), sulfotransferase (SULT), glutathione S-transferase (GST), N-acetyl transferase (NAT), reactive oxygen species (ROS), reactive nitrogen species (RNS), nuclear factor kappa-light-chain-enhancer of activated B cells (NF*κ*B), nuclear factor erythroid 2-related factor 2 (Nrf2), glutathione (GSH), superoxide dismutase (SOD), glutathione peroxidase (GPx), mitochondrial pore transition (MPT), mitochondrial DNA (mtDNA), receptor-interacting serine/threonine protein kinase (RIPK), phosphoglycerate mutase (PGAM), c-Jun N-terminal kinase (JNK), B-cell lymphoma 2 (Bcl2), antigen-presenting cell (APC), and Kupffer cell (KC) [12].

The natures of ADRs are complex. While an individual can have an adverse reaction to a drug, it is difficult to determine the exact cause of the reaction. Many ADRs are caused by the parent drug, the metabolized drug, or byproducts of drug metabolism. The drug metabolism process and the potential triggers for cellular toxicity are illustrated in Figure 1. Drug metabolism relies on an initial transport of drug into the hepatocyte via influx transporters [13]. The drug is metabolized by phase I DMEs, often creating more reactive metabolites [14]. This is followed up with modification via phase II DMEs with a bulkier side chain to deactivate them [14]. The parent drug, reactive metabolites, and heavier products will then all be transported into the bile by efflux transporters compared to those that transport the drug into the cell [13, 14]. Any forms of the drug may produce ROS, which need to be reduced to prevent damage to the cell [15]. Additionally, the drug and its metabolites may conjugate to proteins, forming haptens, which can be presented on the surface of the cell, making it recognized by the immune system as a damaged cell [16]. Any polymorphism that affects drug metabolism, drug transport, antioxidant defense, and immune responses is a potential mechanism associated with increased risk to IDILI.

### 2.1. Phase I Drug Metabolizing Enzymes

Phase I metabolism revolves around modification of the parent drug to create reactive metabolites via formation of alcohol and aldehyde groups (Table 1) [14]. While the parent drug itself has some reactivity, these alcohol and aldehyde functional groups are more reactive with proteins, forming adducts with the proteins that can lead to lymphocyte-signaled apoptosis [18]. Additionally, ROS produced by these functional groups can subsequently damage proteins and DNA via oxidative mechanisms and peroxidize lipids on the cell membrane [19]. These mechanisms can signal caspase-mediated apoptosis and necrosis, respectively [20].

Polymorphisms in phase I DMEs are one of the better characterized mechanisms that are responsible for idiosyncratic reactions. The majority of the proteins that are classified as phase I DMEs are part of the cytochrome P450 oxidase (CYP450) family. The mechanism of metabolism of CYP450 isoforms utilizes a heme to stabilize the intermediate state along with adjacent residues [21]. Each of the different isoforms of CYP450 present in hepatocytes is capable of drug metabolism, but with different specificities for the drugs. There are several isoforms of CYP450 that are clinically relevant in idiosyncratic reactions. Among these isoforms are CYP450 1A2 (CYP1A2), CYP2B6, CYP2C8, CYP2C9, CYP2C19, CYP2D6, CYP2E1, and CYP3A4 [22, 23]. In addition to CYP450 enzymes, flavin monooxygenase (FMO), alcohol dehydrogenase (ADH), aldehyde dehydrogenase (ALDH), monoamine oxidase (MAO), and several peroxidases are involved in phase I drug metabolism [14]. While all of these enzymes play roles in drug metabolism, the majority of incidences of ADRs are due to mutations in the phase I DME genes which result in different polymorphisms of the affected proteins that have altered activity of drug metabolism [22, 24].

For example, CYP1A2 metabolizes many drugs, including caffeine, clozapine, and fluvoxamine [22]. In addition, CYP1A2 assists in the metabolism of bilirubin and several hormones [25]. Two polymorphisms of CYP1A2 have been found to affect metabolism. The CYP1A2^*∗*^1C polymorph is found to decrease caffeine demethylation, while the ^*∗*^1F polymorph is found to increase demethylation [22, 25].

The CYP2 family contains six enzymes with clinically relevant polymorphisms. CYP2B6 metabolizes methadone, cyclophosphamide, and nevirapine [26]. The most common variant of this DME is the ^*∗*^6 allele, which is present in 15–60% of the population (variation is based on ethnicity and race) [22, 27]. This allele and the ^*∗*^18 allele both show decreased activity compared to the wild type 2B6 allele [22, 27]. CYP2C8 mediates the modification of several anticancer, antidiabetic, and antimalarial drugs, including paclitaxel, troglitazone, and amodiaquine [28]. In CYP2C8, the ^*∗*^2 form and ^*∗*^3 form both impact paclitaxel clearance and turnover [29, 30]. CYP2C9 metabolizes an even broader range of drugs than CYP2C8, and several polymorphisms are known to either reduce or eliminate enzyme activity [22, 31]. CYP2C19 metabolizes proton pump inhibitors and several antidepressants [22, 24]. All of the mutant forms, excluding ^*∗*^17, completely eliminate the activity of the enzyme [32]. Ironically, the CYP2C19^*∗*^17 form increases the metabolism of omeprazole, while eliminating the metabolism of the same drugs targeted by wild type CYP2C19 [22, 32]. CYP2D6 metabolizes about 25% of all known drugs, including tricyclic antidepressants (TCAs), selective serotonin reuptake inhibitors (SSRIs), and tamoxifen [22, 33]. There are over one hundred different variants of CYP2D6, and all of the clinically relevant variations show decreased or lack of metabolism by CYP2D6 [33, 34]. Finally, CYP2E1 metabolizes a variety of different drugs, including antitubercular compounds, alcohol, and anesthetics [35, 36]. However, there are mutant genotypes of CYP2E1 that have reduced metabolism of antitubercular drugs [22, 35, 37].

The CYP3A family of CYP450s are the most abundant CYP450 isoforms, metabolizing close to sixty percent of known drugs [38]. Of these, CYP3A4 metabolizes the most drugs, though it has some overlapping activities with CYP3A5 and CYP3A7 [22, 38]. All of the polymorphisms that affect CYP3A4 and CYP3A5 activity either severely reduce or eliminate the activity, while the only known polymorphisms that affect CYP3A7 appear to increase the enzyme's activity [22, 38, 39]. Interestingly, most individuals do not carry a functional CYP3A5, but when it is present, it is responsible for one-third of the CYP3A activity [22, 38, 39].

The other major class of phase I DMEs that have polymorphisms that create susceptibility to ADRs is flavin monooxygenase (FMO). FMO catalyzes the oxygenation of many compounds, particularly at nitrogen sites [40]. FMO3 is both the most abundant of all the isoforms of FMO and the isoform with the most known clinically relevant polymorphisms [40]. Loss of function of FMO3 results in trimethylaminuria, a condition in which the body cannot break down trimethylamine, leading the individual to develop a naturally “fish-smelling” body odor and leaving the individual more susceptible to liver injury [41].

The susceptibility to IDILI due to dysfunction of phase I drug metabolism stems heavily from race. The ^*∗*^1C variant of CYP1A2 is more commonly found in Japanese populations, while the ^*∗*^1F variant appears more frequently in Caucasians [25]. 2B6 allelic expression is heavily tied to race, with the ^*∗*^6 variant and ^*∗*^18 variants most commonly expressed in New Guineans and Japanese, respectively [22, 42]. The ^*∗*^2 allele of CYP2C8 is more common in African populations, while the ^*∗*^3 allele is more common in Caucasian populations [43]. In CYP2C9, the ^*∗*^2 and ^*∗*^3 alleles are more common in Caucasian populations, while the majority of other mutations that reduce CYP2C9 activity are most commonly found in African populations [43, 44]. All of the clinically relevant polymorphisms of CYP2C19 have varying frequency that depends on race, with the ^*∗*^2 form being the most prevalent of the polymorphisms that is found in Caucasians [22]. In the CYP3A family, CYP3A4 does not have significant polymorphisms that are race dependent, but wild type CYP3A5 is more commonly found in African descent and less in Caucasian descent [22, 45]. Additionally, some European populations have a 20% incidence of a double null mutation in FMO3 [22].

### 2.2. Phase II Drug Metabolizing Enzymes

Phase II DMEs modify the drugs following phase I DME modification of the parent drug. The action of phase II DMEs is to replace the reactive aldehyde and alcohol functional groups with less reactive and larger functional groups to direct the drug towards clearance from the body. Phase II DMEs broadly consist of uridine 5′-diphospho-glucuronosyltransferases (UGTs), sulfotransferases (SULTs), glutathione S-transferases (GSTs), and N-acetyltransferases (NATs). All of the phase II DMEs utilize cofactors to conjugate to reactive metabolites to make ready for clearance. While the parent drug and drugs metabolized by phase I and phase II DMEs can all be cleared, clearance of phase II metabolized drugs provides the most stable form of the drug and has a general tendency to lead away from cholestasis and drug-induced liver injury (DILI) [21, 22].

UGTs represent the most diverse class of phase II DMEs. UGTs utilize uridine diphosphate glucuronic acid (UDP-GA) as a cofactor for glucuronidation [46]. Glucuronides are more stable than the reactive intermediates created by phase I DMEs but are significantly less stable than drugs metabolized by other phase II enzymes, particularly reactive acyl glucuronides [46]. There are twenty-four known isoforms of UGT: nine belong to the UGT1 family and fifteen belong to the UGT2 family [46]. Of all of the UGT isoforms, UGT1A1 has the most clinically relevant polymorphisms, owing to the fact that UGT1A1 metabolizes vast classes of chemotherapeutics and bilirubin [47]. The UGT1A1^*∗*^6, ^*∗*^28, ^*∗*^33, and ^*∗*^34 mutations all result in decreased glucuronidation [48]. Having the UGT1A1^*∗*^28 variant results in Gilbert's syndrome and UGT1A1^*∗*^33 or UGT1A1^*∗*^34 results in Crigler-Najjar syndrome, both of which are characterized by hyperbilirubinemia [22, 48]. Certain polymorphisms in UGT2B7 have potentially led to susceptibility to diclofenac induced liver injury, though no other UGTs polymorphisms have been found to lead to IDILI [49].

SULTs are found in the cytosol and perform a sulfation reaction using 3′-phosphoadenosine-5′-phosphosulfate (PAPS) as a cofactor [50]. There are twelve known human SULT enzymes, with SULT1 and SULT2 existing as the most common SULT families in humans [51]. SULT1A1 and SULT1A2 have the greatest number of clinically significant polymorphisms of any of the SULTs [51, 52]. SULT1A1 sulfonates 4-hydroxy-tamoxifen, which actually increases the efficacy of individuals carrying the wild type SULT1A1^*∗*^1 form [52]. This is decreased with individuals carrying the SULT1A1^*∗*^2 form [52].

GSTs have several distinct polymorphisms that carry the potential for idiosyncratic hepatotoxicity. GST conjugates glutathione to drugs for easy clearance [53]. This family of phase II DMEs consists of seven subfamilies denoted by the drugs that they metabolize [54]. The GSTT1 and GSTM1 double null phenotype has been found to lead to troglitazone induced liver injury [55]. GSTP1 is a common metabolizing enzyme of chemotherapeutics, and several polymorphisms have been associated with increased risk of leukemia or susceptibility to chemotherapeutic injury [22].

Humans contain two isoforms of NAT: NAT1 and NAT2 [56]. Both isoforms of NAT function to acetylate parent drugs and reactive metabolites using acetyl coenzyme A (CoA) as a cofactor [56]. Substrates for NAT1 include p-aminobenzoic acid (PABA) and p-aminosalicylic acid (PAS), while substrates for NAT2 include isoniazid, hydralazine, and sulfonamides [22]. While there are approximately twenty-five known polymorphisms of NAT, mutations in NAT2 appear to have a more dramatic effect on acetylation reactions [22]. Slow acetylation has been observed in the NAT1^*∗*^14 and ^*∗*^17 phenotypes, but isoniazid toxicity has been observed in people with null phenotypes of NAT2, and increased toxicity potential towards amonafide was observed in higher NAT2 activity [22].

Incidents of polymorphisms of phase II DMEs have been found to be race dependent. Slow acetylation from NAT2 is found to occur in about half of Caucasians, but only 10% of Japanese [57, 58]. Incidences of polymorphisms in SULT2A1 are significantly higher in people of African descent, but these polymorphisms have not been found to have clinical relevance [45]. Additionally, SULT1A1 polymorphisms that affect activity are found to be represented in different ethnic groups at different frequencies [52]. UGT1A1^*∗*^6 is common in people of Asian descent, while 1/3 of Caucasians and a significant portion of people of African descent are carriers of the UGT1A1^*∗*^28 gene [48, 59]. Additionally, the wild type variant of GSTP1 is present in 60–90% of the population; a statistic that is race dependent [53].

### 2.3. Antioxidant Enzymes

**Figure 2:**
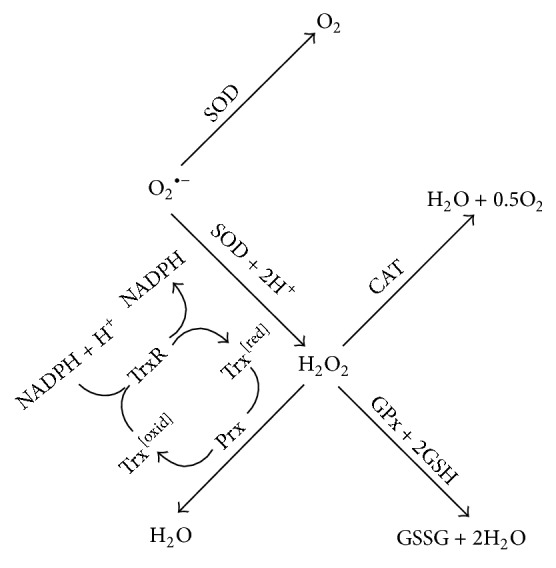
Mechanisms of mediating ROS within the liver. Abbreviations are used as follows: superoxide dismutase (SOD), catalase (CAT), peroxiredoxin (Prx), thioredoxin (Trx), thioredoxin reductase (TrxR), glutathione peroxidase (GPx), glutathione (GSH), and glutathione disulfide (GSSG).

Antioxidant enzymes play a key role in the prevention of cellular injury by reactive ROS (Figure 2). ROS that exist inside the cell include superoxide anion (O_2_^∙−^), hydrogen peroxide (H_2_O_2_), and hydroxyl radical (OH^∙^) among others [60, 61]. A cell's defense to oxidative injury includes the abundance of glutathione (GSH), as well as antioxidant enzymes superoxide dismutase (SOD), catalase (CAT), glutathione peroxidase (GPx), peroxiredoxin (Prx), and thioredoxin (Trx) (Figure 2) [62, 63]. GSH, as a relatively small peptide, can rapidly react with ROS to protect the cell from oxidative injury. However, once approximately 90% of the GSH is depleted within a cell, its susceptibility to oxidative death increases significantly [64, 65]. Thus, the roles of these enzymes in the reduction of ROS are critical to cell survival of oxidative stress.

The antioxidant enzymes within the hepatocytes will generally exist in the mitochondria or the cytosol. However, many of the effects of ROS and the malfunctions of antioxidant enzymes can be observed in mitochondrial behavior. During a hepatotoxic event, mitochondria undergo an event known as mitochondria permeability transition (MPT) [15]. In MPT, stress causes mitochondria pores to swell, allowing passage of solutes of up to 1500 Da, including ROS, Ca^2+^ efflux, and the influx of potentially reactive metabolites [15]. Subsequent events of this include decreased levels oxidative phosphorylation, mitochondrial depolarization, swelling, and subsequent death of the cell by either apoptosis via cytochrome c release or necrosis via ATP depletion [66–68]. Both mechanisms of cell death have been observed to be caused by various hepatotoxicants. Though necrosis is generally found in more cases of DILI, apoptosis is heavily tied with the mechanisms related to immune mediated hepatotoxicity as the role of cytokines in inflammation can exacerbate much of the oxidative stress occurring within the cell [69, 70]. By mitigating ROS, antioxidant enzymes can decrease the frequency of this occurrence.

The mechanisms of these antioxidant enzymes serve to either convert more reactive ROS to H_2_O_2_ or convert all ROS to H_2_O. The enzymatic antioxidants in the body all contain a metallic core to stabilize and catalyze the conversion of ROS to H_2_O. In the case of SOD, the metals at the core can be copper and zinc or manganese for humans, where CuSOD and ZnSOD are in the cytosol or are extracellular, and MnSOD is in the mitochondria [71]. SOD generally converts superoxide anion and water to O_2_ and hydrogen peroxide [71]. Humans contain three isoforms of SOD: SOD1, SOD2, and SOD3. SOD1 is a dimer located in the cytosol, SOD2 is a tetramer located in the mitochondria, and SOD3 is an extracellular tetramer [71]. SOD1 and SOD2 are the more clinically relevant isoforms in regard to idiosyncratic hepatotoxicity since SOD3 is extracellular and affects all organs. Specifically, organisms homozygous for a deletion in SOD2 die due to oxidative damage of the liver, while those heterozygous for functional SOD2 are still at risk for injury [62]. Additionally, people who are heterozygous for either SOD1 or SOD2 deletions are at increased risk for hepatocellular carcinoma [62].

GPx is another enzyme that plays an important role in mitigating oxidative stress, but if deleted may also make an individual more susceptible to hepatotoxicity. GPx serves to catalyze conversion of hydrogen peroxide to water by creating a sulfide bond between two GSH molecules, creating glutathione disulfide (GSSG) [72]. This enzyme works in conjunction with glutathione reductase (GR), which reduces GSSG to GSH via oxidation of NADPH [72, 73]. GPx uses a selenium core to assist in the catalysis. There are eight known isoforms of GPx (denoted GPx1–8), but the two isoforms most involved in hepatoprotection are GPx1 and GPx4 [62, 73]. GPx1 exists in the cytoplasm and mitochondria, while GPx4 is uniformly distributed throughout the cell [62]. Besides converting peroxides to water, GPx also functions in removing peroxides from lipids. Most deletions in GPx have a greater impact on lipid peroxidation than in standard DILI, and fatty liver disease is present in individuals with less functional GPx. Mutations in GPx1 have been shown to potentially induce cholestasis and toxicity due to drug accumulation in the bile [62]. GPx4 mutations generally affect bone tissue more significantly, but there are impacts to liver tissue as well [62].

Among other antioxidant enzymes of note are CAT, Prx, and Trx. CAT is a heme-based tetramer protein that catalyzes the conversion of hydrogen peroxide to water within the peroxisome of the cell [74]. While CAT does play an important role in reducing ROS, deficiencies in CAT do not have correlation with increased risk of idiosyncratic hepatotoxicity, nor is it considered to put an individual at risk for other diseases [62].

Unlike the aforementioned enzymes, Prx, Trx, and thioredoxin reductase (TrxR) do not catalyze the reduction of ROS using metals. Instead, the mechanism relies on interactions with sulfide bonds in cysteine residues on Prx [75]. Prx and Trx work in conjunction with each other, where Prx converts hydrogen peroxide to water. Then, Trx reduces the oxidized form of Prx, and Trx is itself reduced by thioredoxin reductase (TrxR), coupling the oxidation of NADPH to NADP [75]. There are six isoforms of Prx that can be localized to the mitochondria, cytosol, peroxisome, or extracellular space. Deletions in Prx isoforms do not seem to affect hepatotoxicity, though circulating erythrocytes are affected by mutations in Prx. Deletions in any of the genes that code for Trx are lethal, and studies have shown that mice with elevated levels of Trx live longer. Additionally, a recent study has suggested that deletions in genes coding for TrxR1 show to put organisms at greater risk of DILI [76].

The most prevalent cause for susceptibility to ADRs due to problems with antioxidant enzymes is age. The suspected reason for this age related susceptibility is the general decreased ability of cells to deal with oxidative stress. The mitochondria itself is more likely to break down and functions with antioxidant enzymes in the mitochondria, including SOD2 and GPx1 [62]. Additionally, evidence suggests that higher levels of antioxidant enzymes can slow down the body from physiologically aging. This decreased level of antioxidant activity upon aging is a reason suspected for why older patients are at greater risk for experiencing ADRs. In addition to age, postmenopausal females are at a higher risk for DILI than premenopausal females and males that are older than fifty [62]. The hypothesis behind this is that higher levels of muscle tone (particularly lean muscle) promote antioxidant activity. While muscle does degrade with age, women are more susceptible to this injury due to lower initial muscle tone.

### 2.4. Hepatic Transporters

**Figure 3:**
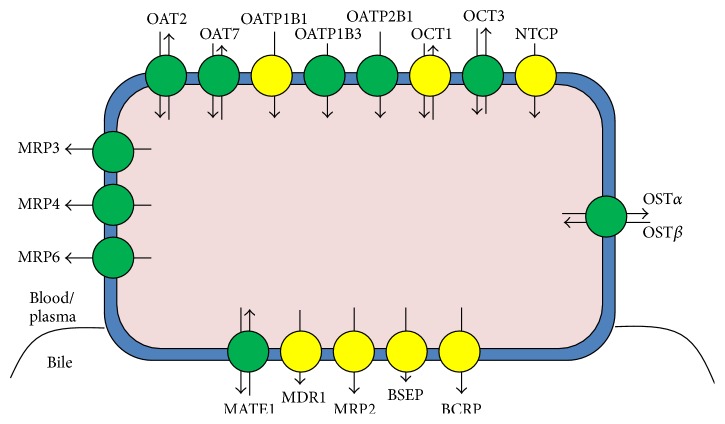
Transporter mechanisms on the cell membranes of hepatocytes. Proteins that are labeled yellow have known clinically relevant polymorphisms, while green labeled proteins do not have known clinically relevant polymorphisms. Abbreviations are used as follows: organic cation transporter (OCT), organic anion transporter (OAT), organic anion transporting polypeptide (OATP), Na^+^-taurocholate cotransporting polypeptide (NTCP), breast cancer resistance protein (BCRP), bile salt export pump (BSEP), multidrug resistance protein (MDR), multidrug resistance-associated protein (MRP), organic solute transporter (OST), and multidrug and toxin extrusion protein (MATE) [13].

Drug transporter proteins are crucial for clearance of reactive metabolites in the liver, kidney, intestine, and brain (Figure 3) [77]. Hepatic transporters can be classified by mechanism of action and by location on the hepatocyte cell membrane. Transport of drugs into or out of the cell can be governed by the ATP-binding cassette (ABC) transporter family of proteins or the solute carrier (SLC) family of proteins [13]. Generally, SLC proteins are considered to be influx transporters [78], moving drugs from the plasma into the cell. ABC transporters are efflux transporters, moving metabolized drugs from within the cell into the bile [79–81].

The transporter families of greatest clinical relevance within the hepatocyte include organic anion transporting polypeptides (OATPs), organic cation transporters (OCTs), multidrug resistance proteins (MDRs), breast cancer resistance protein (BCRP), the bile salt export pump (BSEP), and multidrug resistance-associated proteins (MRPs) [82, 83]. The OCTs and the OATPs are part of the SLC class of transporter proteins, relying on coupled cation/anion transport, as a mechanism of facilitated diffusion down a concentration gradient [82]. Generally, these proteins are characterized as having twelve transmembrane hydrophobic domains that stabilize its structure within the cell membrane. Both classes of proteins function to carry drugs into the cell [14]. While OCT1 is an important liver influx transporter, it is also found within the intestine, so idiosyncratic reactions can occur in both organs [13, 24]. The OATP1B subfamily of proteins (specifically OATP1B1 and OATP1B3) is strictly found in the liver, making it an ideal protein to study for strict hepatotoxicity. Additionally, the Na^+^-taurocholate transporting peptide (NTCP) is another SLC protein that plays a role in influx transport that is localized to the liver [77, 84]. Of all the influx transporters, OATP1B and NTCP are found to have the greatest effect on drug transport [77, 84].

Idiosyncratic reactions are not significantly associated with the SLC transporters. However, OCT1 and OATP1B1 are both found to have clinically relevant polymorphisms. People with defective OCT1 have trouble transporting cationic substrates of metformin [85], while mutations in OATP1B1 have been shown to cause increases in the accumulation of sulfoconjugated troglitazone [13, 24, 86]. Additionally, OATP1B1 mutations have been implicated with increased susceptibility to hyperbilirubinemia [87].

MDRs, BCRP, BSEP, and MRPs are all efflux transporters of the ABC family with clinical relevance in hepatotoxicity. MDR1 (also known as P-glycoprotein) transports a significant amount of xenobiotics and biological compounds into the bile [88–90]. While MDR1 can be found in many tissues, it also has many substrates, making it an important efflux transporter [13]. Additionally, MDR3 is strictly found in hepatocytes, working with ATP8A1 to regulate the transport of phospholipids across the cell membrane [91]. BCRP plays roles in both porphyrin transport and secretion of vitamins in breast milk [92]. While this protein is also found in many cell types, porphyrin release is the most common function found in hepatocytes [92, 93]. BSEP is a protein localized to hepatocytes that functions in the transport of bile salts into the bile canaliculi [94]. As a result, BSEP is an important regulatory in bile flow. Finally, MRP2, 3, 4, and 6 individually act as efflux transporters of hepatocytes, but only MRP2 conducts xenobiotics into the bile [77].

While mutations can occur in both families of transporters, the mutations affecting ABC transporters are considered to be more clinically relevant. Mutations in ABC transporters mean that metabolized drugs cannot leave the hepatocytes, leading to impaired canalicular bile flow. This generally manifests in the form of cholestasis and fatty liver disease [94, 95]. MDR1, BSEP, BCRP, and MRP2 all have polymorphisms that have been associated with idiosyncratic ADRs. Mutations in MDR1 have been implicated in efflux transport of verapamil [89]. Altered expression of BCRP has affected patients taking anticancer drugs or weight loss drugs, including gefitinib, irinotecan, topotecan, and diflomotecan [24, 96]. Mutations in BSEP have been implicated in early onset hepatocellular carcinoma, along with an increase in susceptibility to cholestatic injury from carbocyclic compounds with aromatic rings [97]. Generally, inhibition of BSEP has been correlated with the incidence of cholestatic liver disease [98, 99]. MRP2 mutations can also result in increased susceptibility to hyperbilirubinemia, as well as increased susceptibility to injury due to methotrexate [24, 100] and pravastatin [24, 101].

Age and race both play a significant role in the risk for ADRs from variations in the expression of transporter genes. Increasing age can reduce the expression of several efflux transporter proteins. In particular, mRNA levels of MDR1 are decreased in elderly patients. The expression of MDR1 is generally heterogeneous, and this expression becomes even more pronounced with age [102, 103]. Additionally, OATP1B1 has about fourteen known mutations that have been found to exist within individual populations of people from either European, African, or Asian descent [13]. Frequent polymorphisms of OATP1B1 found in Caucasians include OATP1B1^*∗*^1b and OATP1B1^*∗*^4, but mutations resulting in OATP1B1p.L193R show decreased transport compared to the other forms of OATP1B1 [13]. Individuals with the OATP1B1^*∗*^15 haplotype, which is found in Japanese people, have general reduced transport activity of OATP1B1 [13, 92]. MRP2 mutations found in Korean people have also been found to increase the susceptibility to IDILI by herbal medicines [24].

### 2.5. Immunological Mechanisms

Immunological mechanisms towards drug reactions represent a diverse set of idiosyncratic reactions. Livers have a resident set of macrophages called Kupffer Cells (KCs), which initiate inflammation in the liver after drug exposure [61, 104]. Additionally, the liver is permeated with small populations of other lymphocytes, including other macrophages, T cells, B cells, and natural killer (NK) cells [3, 16, 70]. Owing to the liver's role in detoxification, these cells are necessary for developing foreign body responses.

**Figure 4:**
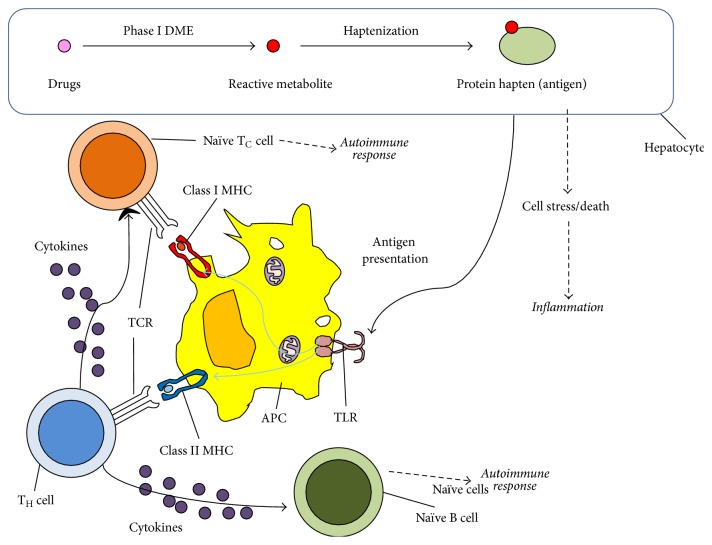
Simplified mechanisms of inflammation signaling caused by adverse drug reactions (ADRs) in the liver. Abbreviations are used as follows: toll-like receptor (TLR), major histocompatibility complex (MHC), T cell receptor (TCR), drug metabolizing enzymes (DMEs), antigen-presenting cell (APC), cytotoxic T lymphocyte (T_C_ cell), and helper T lymphocyte (T_H_ cell).

During drug metabolism, damaged proteins may be expressed on the surface of hepatocytes. Lymphocytes can initiate and regulate inflammation by recognizing drugs and hepatocytes that have metabolized drugs as foreign bodies, either via recognizing the drug in solution or by recognizing damaged proteins on the surface of hepatocytes [61, 105] (Figure 4). During long-term drug exposure, a healthy liver may experience some inflammation that subsides with time. However, ADRs can manifest with significant inflammation in the liver, leading to drug-induced autoimmune hepatitis (DIAIH) or acute liver toxicity [70, 106]. Generally, this mechanism has been considered to be the “failure-to-adapt” model [10]. This model assumes that injury occurs after long-term exposure to drugs and is regulated by the levels of cytokines present during the adverse reaction.

IDILI associated with the failure-to-adapt model assumes the dysfunction in the expression of cytokines. In standard inflammation, a subset of macrophages called M1 macrophages (or, in the case of the liver, KCs) phagocytoses antigens derived from dead or dying cells called damaged-associate molecular patterns (DAMPs) before presentation on major histocompatibility complex (MHC) [107]. In addition to KCs, circulating macrophages, dendritic cells (DCs) neutrophils, and subsets of B and T cells with innate activity can respond to the presence of antigen [107]. Macrophages can present on MHC I or MHC II, which will either lead to the initiation of a cellular immune response by the CD8+ T cytotoxic (T_C_) cells, or a humoral immune response by T helper (T_H_) cells [107]. T_H_1 cells, a variant of T helper cells, also help activate T_C_ cells in a delayed hypersensitivity response via secretion of interferon gamma (IFN-*γ*) and tumor necrosis factor alpha (TNF-*α*) [108]. Another subset, T_H_17 cells, promotes inflammation by secreting interleukin 17 (IL-17) cytokines [109]. In a healthy individual, this inflammation subsides via activity of T regulatory (T_reg_) cells, another T_H_ cell subset that secretes regulatory cytokines, including IL-10 and transforming growth factor beta (TGF-*β*) [108].

The deregulation of cytokines is a potential mechanism for IDILI. In inflammation-based liver injury, the excess of proinflammatory cytokines and the reduction of regulatory cytokines cause general cell death within the liver rather than targeting the liver cells that express damaged motifs [110, 111]. Inducing expression of inflammatory cytokines IL-1*α* and the use of anti-TNF-*α* antibodies increase the susceptibility to DILI [108]. IL-1*α* is proinflammatory, whereas TNF-*α* is regulatory, so the absence of antibodies against TNF-*α* actually increases the chance for inflammation. In addition, IFN-*γ* and IL-1*α* both appear to induce inflammation during acetaminophen-induced liver injury [104, 108]. It has also been found that mutations in anti-inflammatory cytokines IL-4 and IL-10 can increase the risk of diclofenac induced liver injury in humans [112, 113]. While individual mutations can increase susceptibility to injury, double mutations provide an even greater risk to IDILI [11, 114]. Additionally, the source of cytokine deregulation is debated, as mutations can either affect master transcriptional regulators [115] or the actual cytokine [116]. This inflammation deregulation can be found in nonsteroidal anti-inflammatory drugs (NSAIDs) [116, 117] and antibiotics [69, 118].

While KCs are the predominant lymphocyte in the liver, there is a belief that deregulation often stems from defects in T_H_17 cells, leading to autoimmune responses [109]. T_H_17 deregulation has been implicated in other autoimmune diseases, including juvenile diabetes, Crohn's disease, multiple sclerosis, and rheumatoid arthritis [109]. It has been found that levels of proinflammatory cytokines IL-17 and IL-22 are elevated in the presence of penicillamine [114, 119]. In addition, the level of circulating T_H_17 cells is also increased, corresponding with the increases in IL-17 and IL-22 [114]. T_H_17 cell production is induced by IL-6 secretion of macrophages, which is pleiotropic [109]. Thus, there is significant interplay between the types of cells in the immune system and the potential for idiosyncratic drug reactions.

Another potential mechanism of IDILI is that human leukocyte antigen (HLA) haplotype will affect the recognition of the immune system of cells presenting damaged motifs. HLA codes for the MHC in humans. In adults, the HLA-A, HLA-B, and HLA-C genes govern the structure of MHC I, while HLA-DP, HLA-DQ, and HLA-DR govern the structure of MHC II [120]. While there is one gene for each of the MHC I coding regions, there are eight genes responsible for MHC II [120]. Individually, this leaves 1000–2000 different allotypes for each of the HLA-A, -B, and -C genes and 2–860 allotypes for each of the eight -D genes [120]. This yields a total of 1.7 billion haplotypes for MHC I and 10^15^ haplotypes for MHC II.

Generally, HLA polymorphisms associated with increased susceptibility to IDILI are found on the MHC II locus. This means that signaling from CD4+ T_H_ cells is directly affected, as these cells have direct interaction with MHC II. Additionally, because of the significant role of T_H_ cells in regulatory signaling, there are effects seen in the behavior of other lymphocytes, including T_C_ cells and KCs. The majority of known polymorphisms associated with HLA are found on HLA-DRB1, though there is a significant presence of polymorphisms affecting HLA-DQ as well [24, 121, 122]. While most of these polymorphisms are associated with increased disposition towards cholestatic liver injury, there are at least three known polymorphisms associated with increased toxicity towards amoxicillin [121, 122]. There are a set of mutations in HLA-DQ that predispose towards lumiracoxib-related liver injury [123]. Additionally, HLA-B has at least one known polymorphism to increase susceptibility to flucloxacillin [124, 125], and others that are more predisposed towards antiepileptic drugs [111]. Owing to the fact that HLA-DQ and HLA-DR polymorphisms code for polymorphisms in MHC II, it is possible that many of the deregulated cytokines are as a result of indirect effects from MHC II with altered functionality.

IADR predisposition via possible immune-based mechanisms can be found as a function of gender, age, race, and current immune state. Often, those that are immune compromised, such as people with autoimmune illnesses affecting inflammation will be more at risk for IDILI due to the dysfunction of cells that govern both mechanisms. It has been found that women with breast cancer with the HLA-DQA1^*∗*^02:01 are more at risk to develop an IADR to certain breast cancer therapeutics than women with breast cancer with a different HLA haplotype [126]. Japanese are more at risk for HLA-mediated ticlopidine IDILI [127]. Additionally, age plays a significant role in cytokine-mediated ADRs, as DNA is more susceptible to mutation and autoimmune diseases inducing inflammation become overly prevalent [106, 109, 114].

## 3. Assays Detecting Susceptibility for Idiosyncratic Reactions

In clinical trials and in vivo tests, the assessment of ADR potential is developed by use of measuring biomarkers alanine transferase (ALT), aspartate transaminase (AST), and bilirubin [128, 129]. ALT and AST are general biomarkers for protein catabolism, while bilirubin is an indicator of cholestasis and bile flow. While other biomarkers have been developed to assess liver behavior, the combination of elevated ALT, AST, and bilirubin levels is the general indicator for potential of liver injury. The rule for assessing if DILI will occur was developed by Hy Zimmerman: it states that liver injury is likely to occur when an organism is exposed to drugs exhibits five times the upper limit of normal (ULN) for ALT activity, 3 × ULN for AST, and 2 × ULN for total bilirubin (TBL) found in serum [130]. While positively identifying that the markers for Hy's Law correlate with increased risk for DILI, the absence of these markers does not rule out liver injury [128]. Additionally, it is possible that the combination of factors may not even result in injury to the liver. Thus, reliance on other markers to predict and detect injury is necessary for proper drug development.

Developing assays for detecting the potential for IDILI relies on screening individual events. While conventional assays may characterize drug metabolite formation and the general potential for ADRs; very little emphasis has been put on detecting the potential for IADRs. Assays for detecting inflammation in vivo and CYP450 activity assays in vitro have been developed for clinical and industrial use, but very few assays have been developed to predict ADRs in individuals with polymorphisms in transporters, phase II DMEs, or antioxidant enzymes. Here, we will describe assays that can be used for detecting IADRs, with emphasis on in vitro systems, preferably ones that can be used for high-throughput screening (HCS) of potentially toxic compounds (see Table 2).

### 3.1. CYP450 Activity Assays

**Figure 5:**
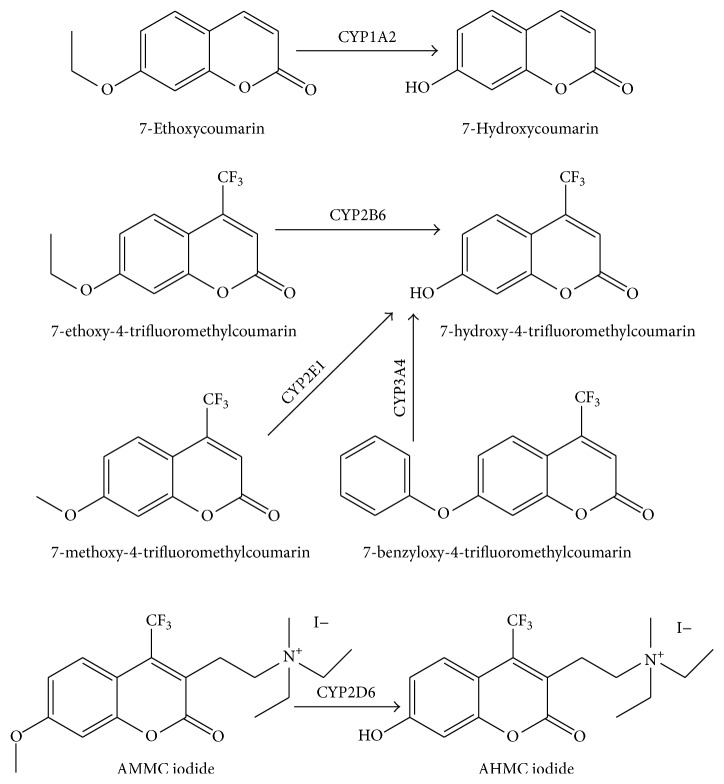
Metabolism of coumarin by CYP450 isoforms. Abbreviations are used as follows: 3-[2-(N,N-diethyl-N-methylammonium)ethyl]-7-methoxy-4-methylcoumarin (AMMC), and 3-[2-(N,N-diethylamino)ethyl]-7-hydroxy-4-methylcoumarin (AHMC).

Measuring individual enzyme activity can provide information on potential mutation of DMEs indirectly. Coumarin-based substrates have been used to determine the activity of various CYP450 isoforms (Figure 5). Each isoform can metabolize a different substrate, though there could be some substrate overlap [23, 131]. For example, 7-ethyloxymethyloxy-3-cyanocoumarin (EOMCC) can be metabolized via CYP1A2, CYP2D6, or CYP2E1, along with other CYP450 isoforms. The listed reactions occur via hydroxylation of the ethoxy or methoxy oxygen on the fluorescent compound. Additionally, all of the metabolites excite at 355 nm, but the HFC emits at a different wavelength than the metabolites formed by CYP1A2 and CYP3A4 metabolism, allowing for two CYP activities to be measured in one well. The drawback to these substrates is that if the cell line is capable of significant phase II metabolism, inhibitors need to be added to the solution to properly assess CYP450 activity.

While CYP2C activity might not be modeled by the coumarin fluorogenic substrates, activity can still be measured using conversion of dibenzylfluorescein (DBF) to fluorescein by CYP2C8 and CYP2C9 [23]. DBF requires low concentrations for drug metabolism and generally gives a strong fluorescent signal when CYP2C9 metabolism is present. Additionally, CYP3A4 activity may be quantified using Luciferin-based luminescent reagents [132].

In addition to standard fluorogenic substrate assays, drug metabolites can be pooled from media and quantified using high performance liquid chromatography (HPLC) and mass spectrometry (MS) [133–135]. The advantage of this is that endpoint activity can be quantified along with cell viability to determine if a therapeutic is potentially toxic to individuals or whole populations. However, this system is generally better suited for endpoint therapeutic studies, and there is great financial cost associated with performing HPLC and MS to quantify CYP450 activity.

In addition to recombinant individual DMEs used, human liver microsomes (HLMs), the centrifuged product of lysed liver tissues, are commonly used to measure a desired activity. Generally, HLMs contain a large concentration of CYP450s and some phase II DMEs such as UGTs, and HLMs can be used to measure the expression levels of CYP450s in the liver tissues from which they were isolated. Walsky and Obach used HLMs to monitor metabolic activity over a range of compounds that can be metabolized by CYP450s [131].

### 3.2. Phase II DME Assays

Specific protein assays that are relevant to IADRs are targeted towards measuring UGT and GST activity. One way to measure UGT activity is the fluorescent substrate 4-methylumbelliferone (4-MU), which is a coumarin derivative that undergoes glucuronidation [136, 137]. UGT's role in clearance of steroids makes steroidal compounds good substrates to assay for UGT specific activity as well [82]. In the case of GST, levels and activity can be quantified via the removal of chlorine from substrate 1-chloro-2,4-dinitrobenzene (CDNB) [138]. While CDNB can be used as a substrate for total GST activity,* p*-nitrobenzyl chloride (NBC), 1,2-chloro-4-nitrobenzene (DCNB), and trans-4-phenyl-3-buten-2-one (PBO) can be used to detect GSTM activity, and 1,2-epoxy-3-(p-nitrophenoxy)propane (EPNP) and dichloromethane (DCM) can be used as substrates for GSTTs [139, 140].

A functional assay can be used to determine idiosyncratic reactions in phase II DMEs in determining the covalent binding level (CBL) [55]. Because drugs that have been only metabolized by phase I DMEs are more reactive than the parent drug or drugs that have been successfully conjugated with bulkier functional groups, computation of CBL can be used to assess either reactive metabolite formation potential or the rate at which the metabolite is not being cleared from the hepatocyte.

Glucuronidation can be quantified using methods such as HPLC and MS [141]. Surendradoss et al. used HPLC and MS to quantify valproyl 1-O-acyl-glucuronide (VPA-G), the phase II metabolized drug of valproic acid [142, 143]. Additionally, diclofenac conjugated to GSH has been measured via HPLC to assay for GST activity [140]. The drawback to using these technologies is that metabolites can only be quantified in relative terms without giving exact values as to the forms of drug present in a solution. This leads metabolite analysis to be performed only for diagnosing an idiosyncratic event rather than determining its severity. For reactive metabolite counts, GSH trapping can be used to quantify the level of oxidative metabolites before being run through HPLC and MS [144]. Additionally, adduct formation of proteins can also be measured using LC-MS, providing insight towards the mechanism of injury [117, 145].

In in vivo models, the GSTT1 and GSTM1 single-null and double null mutation has been thoroughly studied in regard to troglitazone induced ADRs in mice [35, 55]. These particular models are beneficial because there has been shown an additive effect towards generating IDILI with these responses.

### 3.3. Mitochondrial Activity Assays

One of the difficulties associated with determining idiosyncratic reactions with antioxidant enzymes is that very few of the assays can target specific protein functions, and they are designed for general ADRs as mitochondrial dysfunction is often a symptom (rather than the cause) of hepatotoxicity. Due to the fact that ADRs blamed on antioxidant toxicity are associated with proteins found in the mitochondrion, it is the primary indicator for liver functionality tests. Certain assays, such as the cupric ion reducing antioxidant capacity (CUPRAC) assay [146] or the MitoTracker dye [147], can rely on calculating general antioxidant capacity within a cell, or confocal microscopy to examine individual mitochondrial behavior. However, general assays are more geared towards calculating antioxidant potential associated with smaller molecules, including ascorbic acid and tocopherol. Additionally, 2′,7′-dichlorodihydrofluorescein (H_2_DCF) can be used to measure general oxidative stress [148, 149]. However, this too is not specific for idiosyncratic mechanisms.

Mitochondrial dysfunction is a considerable target for drug toxicity screens. For general mitochondrial toxicity assays, measuring mitochondrial membrane potential (MMP) via fluorescent compound tetramethyl rhodamine (TMRM) can be used to quantify apoptosis as MMP depolarization is symptomatic of the issue [148]. Besides MMP, cytochrome c release and variation in the oxygen consumption rate (OCR), which is the turnover of O_2_ during metabolism, may be symptomatic of mitotoxicity [150–153]. MitoTox has been used as a phosphorescent probe for the general measurement of OCR [151–153]. Additionally, Liang et al. managed to purify mitochondria and assay for GPx activity, though the method is not durable for high-throughput applications [138].

Owing to the role of mitochondria in reduction of ROS, antioxidant GSH is an adequate substrate for assessing mitotoxicity. One commonly used compound, monochlorobimane (mBCl), is fluorogenic in the presence of glutathione [154]. This substrate reacts with thiol based compounds, so fluorescence can correlate well with substrate depletion [154, 155]. While GSH acts as a general indicator for oxidative stress, this can be sufficient enough to indicate dysfunction with antioxidant enzymes, or even the general presence of an IADR. Other protocols are able to discriminate between GSH and GSSG based on the GS-GPx oxidation-reduction mechanism [101, 156]. The protocol however is reagent intensive and is not compatible with high-throughput drug screening.

One of the more common mutations studied is a heterozygous SOD2 mouse [117, 154]. A homozygous knockout for SOD2 is lethal to the organism due to SOD2 being the major enzyme responsible for removal of oxidant sources in the cell. Yet, a heterozygous expression of normal SOD2 results in a viable mouse with a decreased ability to reduce intracellular ROS. The SOD2 heterozygous mouse has been used to study cardiac function, aging, and drug metabolism. While significant studies have been done to show the potential for gene knockouts in SOD2, very few studies have been done for GPx1, which has also been shown to have polymorphisms with clinical relevance.

### 3.4. Transporter Assays

In the assessment of transporter activity, BSEP is commonly assayed because its dysfunction is correlated with cholestasis. Common BSEP assays include the vesicular transport assay (VTA) and the ATPase assay [94], both of which rely on the presence of ATP as a to drive the protein function and hence the assay. Because both assays rely on ATP, this makes VTA and the ATPase assay also suitable for ABC transporter proteins. In addition to VTP and ATPase, taurocholate may be used for measuring BSEP transport [157].

Drug uptake and release assays are also available for other compounds and transporter proteins [158]. Owing to the common regulation of MDR1 and CYP3A4 via the pregnane X receptor (PXR), there is significant overlap in compounds that can be used to assay transporter activity [77, 86, 88]. Examples of this are rifampicin and verapamil as inducers and inhibitors of MDR1 activity, respectively [77]. In the influx transporter system, OATP1B1 and OATP1B3 have broad substrate specificity that can be used to measure activity, including rifampicin [159, 160], taurocholate [161], and pitavastatin [82]. For other transporters, MRP2 is inhibited via a fluorescent compound, 5(6)-carboxy-2′,7′-dichlorofluorescein (CDCF) [82, 157, 162]. Additionally, OCT knockouts have been generated in mice, including double knockouts of OCT1 and OCT2 [77].

Flow cytometry can be used to diagnose potential for idiosyncratic reactions via transporter and immune mediated mechanisms. The principle of flow cytometry utilizes cell counting and cell sorting based on immunofluorescent labeling of cell surface markers. Transporter proteins and lymphocytes are ideal detection markers for flow cytometry because they exist on the cell surface and are stainable with fluorescent antibodies. Saab et al. used microvolume flow cytometry to assess the behavior of MDR1 and MRP2 as associated risks with IDILI [89]. By staining for efflux transporters, they were able to detect toxicity potential due to synergistic effects of several known idiosyncratic drugs with known effects to induce inflammation. Perez et al. used flow cytometry to quantify the behavior of several efflux transporters by examining ROS production due to mitochondrial deregulation [90].

### 3.5. Inflammation Assays and Immune System Dysfunction

Cytokine analysis is an effective tool for quantifying ADRs for long-term drug administration. In addition, cytokines can be readily quantified in both serum and media. A common practice is to administer agonists that induce inflammation, such as lipopolysaccharide, (LPS) to activate toll-like receptor- (TLR-) mediated inflammation and apoptosis [163, 164]. Caspase 3/7 activity assays are a common assay used to measure inflammation-mediated apoptosis [19]. Because of the diversity of symptoms associated with IDILI, time dependence is a concern for most clinicians on the response. However, inflammation turnaround can be rather quick compared to other methods of IDILI. The significant drawback to inflammation assays is the complexity. Common methods for quantifying cytokine levels include ELISA and RT-PCR [163]. Due to toxicity occurrence coming from the immune system rather than the liver, multiple cell systems and pathways need to be monitored to determine the dysregulated cytokines.

Yano et al. attempted to look at the complexity of this issue by stimulating inflammation in mouse livers [163]. They added drugs at known concentrations and measured cytokine levels from mouse models to compare with the current elevations in standard liver biomarkers ALT and AST. Additionally, liver inflammation markers were compared in in vitro monocyte cultures with and without the presence of heat-inactivated HLMs by observing gene expression. Ultimately, the results showed a time dependent responsiveness towards cytokine development, with several of the elevated proinflammatory markers (IL-1*β*) clearly elevated within a 24-hour time window. Additionally, a transcription factor that promotes inflammation that is also found in plasma, high-mobility group protein B1 (HMGB1), was only found in high quantities of drugs that are considered hazardous to the liver. However, the biggest take away was the combined effects on the expression of MRP8, MRP9, IL-1*β*, and cryopyrin (NALP3), and receptor for advanced glycation end products (RAGE) would be elevated in the presence of drugs with increased susceptibility to liver injury. All of these markers are direct results of upregulation of the nuclear factor kappa B (Nf-*κ*B) pathway.

There are several other assays besides direct quantification cytokine levels. Master transcriptional regulators may become a target for measuring specific lymphocyte activity [115]. By measuring the levels of master transcriptional regulators, scientists and clinicians have ideas of which cells are proliferating and which pathways are stimulated. Another test that can be used to assess drug toxicity is the lymphocyte transformation test (LTT) [121, 165]. Here, cells proliferate in response to the presence of particular antigens or drugs.

Additionally, flow cytometry can be used to quantify adverse behavior in lymphocytes. It has been used to quantify the presence of KCs and other cells that are involved in inflammation-based toxicity [166], cytokine synthesis during inflammation due to drug allergy [165], and the upregulation of cluster of differentiation 69 (CD69) [165], a biomarker for inflammation. Thus, injury can be assessed from in vivo and in vitro models using flow cytometry.

In addition to drug metabolite quantification, metabolism is a method for specifying immune cell activity. Broadly, lymphocytes can be categorized based on aerobic versus anaerobic glycolysis and whether or not oxidative phosphorylation is taking place. The presence of aerobic glycolysis without oxidative phosphorylation indicates the increased activity of cells often found at the site of inflammation, including neutrophils and KCs.

Cytokine knockouts have been used to monitor the potential for ADRs in mouse and rat models. Common knockouts that have been used to increase susceptibility to IDILI include TNFR1 [108, 167], IL-13 [108, 168], and double knockouts of IL-4 and IL-10 [108, 169]. In addition, IFN-*γ* and toll-like receptor 9 (TLR9) individual knockouts seem to decrease the susceptibility to IDILI [108]. In addition, while the double knockout of IL-4 and IL-10 increases susceptibility to ADRs, having a triple knockout of IL-4, IL-6, and IL-10 decreases susceptibility to IDILI [108, 169].

### 3.6. Genetic Regulation and Clustering

Creating genetic profile maps can give a diversified predictability of IADRs. Focus has been maintained on clusters of simultaneously regulated genes [170, 171] or genes that could potentially contribute towards IDILI [117]. Aside from genetic profiling, only flow cytometry may be able to predict IADRs due to HLA haplotypes [172], providing a necessary function for predicting some mechanisms of IDILI. Clustering in particular is important for assessment of commonly regulated genes and finding mutations within pathways. Targets for clustering analysis include xenobiotic metabolism, bioenergetics, and mechanisms for inflammation and injury [173]. Another target for genomic analysis is study of the peroxisome proliferator-activated receptors (PPARs), which are nuclear receptor proteins that control for transcription of various enzymes involved in drug metabolism [60, 174]. Additionally, the AmpliChip CYP450 Test has been developed to detect CYP450 polymorphisms [175, 176]. While gene regulation can give indication towards missing enzymes or downregulated pathways, clusters do not necessarily directly correlate with protein expression or activity at either the DNA or the RNA level as posttranscriptional modifications and RNA degradation may occur. Even so, models that focus on pathway regulation and gene analysis related to mechanisms of injury can be good predictors of idiosyncratic compounds [170, 177].

### 3.7. Predictive Models

While focus has been given towards in vitro and in vivo test, computational results can rule out a significant amount of drugs that have toxic potential. Of particular importance is the prediction of reactive metabolite formation [178] and how those metabolites may interact with intracellular molecules [31, 179], or looking at drugs that affect major regulatory molecules for drug metabolism [180, 181]. These models, while maintaining importance towards predicting general ADRs, may also be used if certain classes of compounds create toxicity for individuals with any clinically relevant polymorphisms.

## 4. Towards Integrative Platforms for Comprehensive IADR Predictability

While several of the current systems and assays emphasize detection of single mechanisms for hepatotoxicity, many of the current in vitro systems fall short at generating complete individual profiles for predicting IADRs. While in vivo models can be used to generate comprehensive profiles for ADRs and simultaneous events, they generally have poor predictability and are more expensive than in vitro assays [150]. The rest of this review will focus on developing in vitro and ex vivo cellular models that can be used to assess cumulative idiosyncratic potential with applications in diagnosing idiosyncratic potential and drug development.

### 4.1. Controlled DME Expression Systems

Significant advantages of utilizing hepatomas or immortalized cell lines are the well-characterized expression of proteins from these cell lines and the affordability they present for clinical and industrial tests. While hepatocytes contain many of the significant proteins for drug metabolism, maintenance of these cultures is expensive and often the cells will lose their in vivo protein expression hours after being cultured. Thus, controlling the expression of DMEs, transporters, and antioxidant proteins via manipulation of the genome in immortalized cell lines provides a cost-effective and reproducible method for characterizing idiosyncratic potential. Additionally, using cells that either have expressions that can be easily altered or adequately reflect in vivo behavior of hepatocytes is the most important for any in vitro toxicity assay.

Among the most utilized forms of transformable liver cell lines are epithelial cells transformed with SV40 large T antigen (THLE cells). These cells have very low expression of phase I and phase II DMEs, making them ideally suited for transducing genes that express DMEs [182]. Viral transduction can be readily performed on these cells to express the desired DMEs. Kwon et al. successfully transduced recombinant adenoviruses expressing several clinically relevant CYP450 isoforms into THLE-2 cells on a miniaturized platform that is effective at generating dose-response curves for tested therapeutics [183]. Various combinations of DMEs expressed in THLE-2 cells on the chip can be used to potentially simulate systematic compound metabolism and toxicity, including idiosyncratic ADRs [183]. Thompson et al. also used THLE-2 cells transformed with CYP450 enzymes to successfully assess idiosyncratic risk of several drugs [157]. While this approach allows for tunability in enzymatic expression for several of the key DMEs, using compounds or short hairpin RNAs (shRNAs) to inhibit activity of specific DMEs within hepatic cells is also a reliable approach [184].

Besides THLE cells, HepaRG have been used to quantify drug metabolic activity. Unlike most other cell lines, HepaRG cells are derived from progenitor cells and retain much of the activity of in vivo human hepatocyte cultures, including CYP450 activities [136]. While not as easily transformable as the THLE cell expression system, HepaRG cells have some tunability with expression of clinically relevant sources of IDILI [185, 186]. Anthérieu et al. induced differentiation in HepaRG cells towards successfully mimicking hepatocyte behavior, including both phase I and phase II DME activity [187]. As illustrated by Mueller et al., HepaRG cells also have the potential for 3D growth in hanging droplet experiments, and activity of critical enzymes was increased compared to monolayer controls [188]. This is not to limit cells used for viral transduction to affect DME expression, as HepG2 have also been successfully transduced with adenoviral vectors containing DNA coding for the expression of CYP450s [189].

### 4.2. Hepatic Coculture Systems

Liver coculture systems provide the advantage of giving a more complete profile of liver behavior in the presence of drugs. Since the liver contains multiple cell types, the coculture system allows examination of the effects of drugs on different functions. While the liver is primarily composed of hepatocytes, there are other cells present, including several different kinds of lymphocytes, sinusoidal endothelial cells, and stellate cells [190]. In addition, the liver is organized based on proximity to blood vessels. Cells closer the portal vein are more involved in oxidative metabolism, *β*-oxidation of fatty acids, ureagenesis, and gluconeogenesis, whereas cells further from major blood vessels are involved in biotransformation of drugs, glutamine synthesis, lipid synthesis, and glycolysis [191]. As a result, development of models for liver structure in hopes for predicting idiosyncratic ADRs is a complex process.

**Figure 6:**
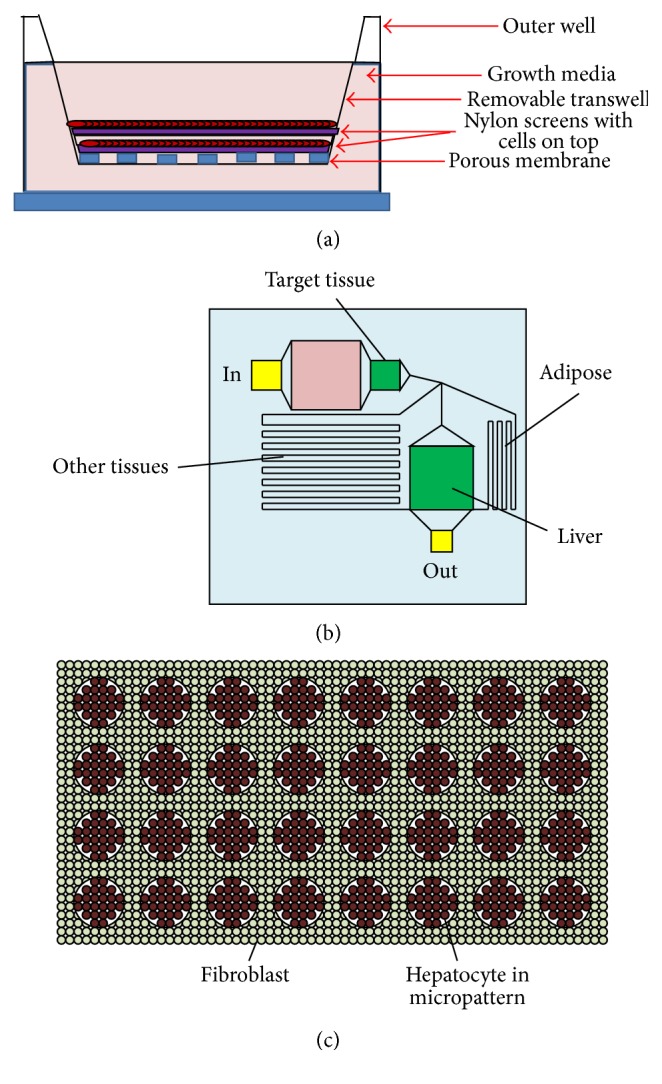
Hepatic cell coculture systems with applications in hepatotoxicity: (a) porous nylon membrane scaffold system [192], (b) the microfluidic system [193, 194], and (c) micropatterned hepatic cocultures [195].

Kostadinova et al. implemented a coculture system by using porous layered nylon scaffolds designed to fit 24-well plates, which allowed the cells to generate their own 3D matrix and growth factors by expansion between the pores (Figure 6(a)) [192]. Additionally, this scaffold had smaller pores to allow for nutrient regeneration and removal. Cells cultured on this scaffold included hepatocytes, stellate cells, KCs, and endothelial cells. Ultimately, this scaffold was successful at maintaining in vivo conditions for normal liver function for eleven weeks and was able to induce proinflammatory cytokine production via addition of LPS to scaffolds. Additionally, CYP450 assays and drug uptake were effectively measured in this system to account for several mechanisms that an affect idiosyncratic responses. While this system utilized a small scale, moderate throughput design, scaling down for 96-well and 384-well plate assays would be difficult due to the maneuverability of the scaffold.

Novik et al. present a coculture system that utilizes microfluidic cocultures of hepatocytes and nonparenchymal cells (NPCs) for clearance studies (Figure 6(b)) [193]. The advantage of this system is that there is tunability in O_2_ parameters and flow rates, which is often missing in conventional high-throughput screens. Novik et al. were able to assess that phase I DME activity was elevated in the presence of flowed cocultures compared to static cultures and cultures with just hepatocytes. Additionally, the bile canaliculi formation was successfully imaged to indicate that transporter activity could be measured. However, there are limits to flow systems for high-throughput detection, making this particular system less advantageous for industrial use.

Several groups have implemented fibroblasts to facilitate the creation of a microenvironment that best mimics the liver (Figure 6(c)). Khetani et al. implemented micropatterned cocultures using fibroblasts [196]. Ultimately, while assays restored some liver functions, there were several false negatives with detecting compound toxicity, owing to the lack of diversity and monolayer culture used for these cells. Wang et al. micropatterned cocultures like Khetani et al., but utilize general stromal cells instead of fibroblasts, measuring their results by quantifying circulating metabolites two and seven days after incubation [197]. While success rates were higher for detecting phase I and phase II metabolites compared to microsome analysis or suspension cultures, toxicity results were accurately predicted only about 70% of the time in two-day cultures and 80–85% of the time in week-long cultures. Thus, cocultures with fibroblasts in the absence of KCs, stellate cells, and endothelial cells may not be ideally suited for drug metabolism studies. More recently, Rose et al. have developed a system using plated hepatocytes cocultured with KCs to model the inflammation response within a 48-well collagen-coated plate [132]. The group was successful in measuring the concentrated immune response as well as CYP3A activity using the previously mentioned luciferin substrate [132].

Disadvantages of coculture platforms involve the complexity of the scaffolds to accurately mimic the in vitro liver environment, as well miniaturization being limited due to the number of cells needed to accurately determine toxic effects. The assay miniaturization is of particular importance with microfluidic platforms and the removable inserts, as this also impacts scalability and cost. Even integrating all cell types on a 96-well plate platform is challenging because decreasing the number of cells limits the interaction observed between different cell types. Thus, size of culture optimization should be considered when designing small scale liver tissues for toxicity testing.

### 4.3. Stem Cells for Modeling DILI

Besides coculture platforms, stem cells have become a larger part of the investigation into mechanisms that cause DILI and ADRs. In particular, the use of induced pluripotent stem cells (iPSCs) can be used to generate the necessary cell types to mimic the adult liver and model disease states. In iPSCs, adult somatic cells are reverted to behaving like embryonic stem cells (ESCs) via introduction of several key transcription factors [198]. While iPSCs require dedifferentiation with subsequent redifferentiation to derive appropriate cell lines, they provide an innovative way to both model liver disease and assess how certain polymorphisms may affect drug metabolism and disposition in vitro. Additionally, human iPSCs (hiPSCs) have a significant amount of commercial availability for applications in liver toxicity testing and have the potential to be used in coculture with hepatocytes and fibroblasts to give an accurate model for predicting potential liver toxicity [199–201].

It is becoming more important that the role of iPSCs is not only to derive hepatocyte-like cells, but also to recapitulate their function and potential polymorphisms to generate accurate models. Szkolnicka et al. have recently demonstrated the ability of redifferentiated iPSCs to express CYP1A and CYP3A DMEs and express similar responses to drugs as compared to cryopreserved hepatocytes cultured in vitro [202]. Individualized toxicity assessments have been completed by various groups as Choi et al. used patient-specific cell lines for individuals deficient in the alpha-1 antitrypsin (A1AT), a protease inhibitor that when absent has been correlated with liver cirrhosis and heptocellular carcinoma [203]. While maintaining levels of expression is an ongoing issue in the field of using stem cells and iPSCs in particular, there has been recent progress made towards having redifferentiated cells express appropriate levels of key DMEs to be used as models in drug metabolism [204].

### 4.4. Precision-Cut Liver Slices

As an alternative to standard in vitro models, precision-cut liver slices can be studied to determine the interactions of various cells in the liver in order to recapitulate conditions that can lead to IDILI. The main advantage of this ex vivo technique is that with all cells present, the goal of coculture is achieved without reconstructing the entire liver from the bottom up. Additionally, most DME and transporter expression are preserved during the toxicity testing [205]. Hadi et al. have demonstrated this use for detecting inflammation-induced liver injury while quantifying the levels of GSH and proinflammatory cytokines in mouse and human-derived liver slices [156, 206]. In addition to ADRs, liver disease states can be modeled, ranging from fibrosis to obesity [207, 208]. The disadvantage of this technique is that because precision-cut liver slices need to be obtained from whole organisms, surgery or sacrifice of specimens is required, and achieving HTS for adequate drug modeling is difficult. While these are issues, precision-cut liver slices do provide an adequate way to model liver disease states.

## 5. Summary

With at least six general causes that have been potentially tied to IADRs, the need for predicting IADRs before in vivo experiments is critical. While individual drugs that might induce inflammation or have variant metabolism based on CYP450 expression are generally predicted before clinical trials, less focus has been give towards other potential theories regarding IADRs. There are currently assays available for high-throughput applications that can be used for predicting certain IADRs based on the individually described mechanisms. Simulating simultaneous idiosyncratic events requires platforms that can control the behavior of multiple events at once, or ones that can mimic the in vivo environment of the human liver. Future research should be focused on developing high-throughput platforms and utilizing assays that can predict ADRs in vitro.

## Figures and Tables

**Table 1:** Examples of drug metabolizing enzymes (DMEs) [17].

Classification	Enzymes	Overall reactions
Oxidative DMEs (Phase I reactions)	Cytochrome P450 (CYP450)	*Carbon oxidation* RH + O_2_ + NADPH + H^+^→ ROH + H_2_O + NADP^+^
	Flavin-containing monooxygenase (FMO)	*N (or S) oxidation* R-NH-R′ + O_2_ + NADPH + H^+^→ R-NOH-R′ + H_2_O + NADP^+^
	Monoamine oxidase (MAO)	*Oxidative deamination* R-CH_2_NH_2_ + O_2_ + H_2_O → R-CHO + H_2_O_2_ + NH_3_
	Alcohol dehydrogenase	*Alcohol oxidation* R-CH_2_OH + NAD^+^→ R-CHO + NADH + H^+^
	Aldehyde dehydrogenase	*Aldehyde oxidation* R-CHO + NAD(P)^+^ + H_2_O → R-COOH + NAD(P)H + H^+^
	Aldehyde oxidase	*Aldehyde oxidation* R-CHO + O_2_ + H_2_O → R-COOH + H_2_O_2_

Conjugative DMEs (Phase II reactions)	UDP-glycosyltransferase (UGT)	*Glucuronidation* R + UDP-glucuronic acid → R-glucuronide + UDP
	Glutathione S-transferase (GST)	*Glutathione conjugation* R + GSH → GS-RR-X + GSH → GS-R + HX
	Sulfotransferase (SULT)	*Sulfation* R-XH + PAPS → R-SO_4_ + phosphoadenosine + H^+^
	N-Acetyltransferase (NAT)	*Methylation* R-NH_2_ + CoA-S-COCH_3_→ R-NCOCH_3_ + CoA-SHR-NHOH + CoA-S-COCH_3_→ R-NHOCOCH_3_ + CoA-SH

Xenobiotics (R), *β*-nicotinamide adenine dinucleotide phosphate (NADP), *β*-nicotinamide adenine dinucleotide (NAD), uridine 5′-diphosphate (UDP), glutathione (GSH), 3′-phosphoadenosine 5′-phosphosulfate (PAPS), and coenzyme A (CoA).

**Table 2:** Assays used for determining IDILI potential.

Mechanisms	Proteins	Relevant assays
Phase I DMEs	CYP450	Coumarin metabolism Fluorescein (CYP2C9) Human liver microsomes (HLMs) High pressure liquid chromatography (HPLC)/Mass spectrometry (MS)

Phase II DMEs	UGT	Coumarin metabolism
	GST (GSTT1 and GSTM1)	CDNB, NBC, DCNB, EPNP, DCM, PBO GSTT1/GSTM1 single and double knockouts
	UGT/GST/NAT/SULT	Covalent binding level (CBL) HPLC/MS

Antioxidant enzymes	GPx1/SOD2	Monochlorobimane (mBCl) Oxygen consumption rate (OCR) Fluorescein Tetramethyl rhodamine (TMRM) Cytochrome c release SOD2^+/-^ mice

Transporters	BSEP	Vesicular transport assay (VTA) ATPase assay
	MRP2	Fluorescein
	MDR1/MRP2	Flow cytometry
	OCT	OCT1 and OCT2 knockouts
	BSEP/BCRP/MDR/MRP/OATP/OCT/NTCP	HPLC/MS Drug uptake assays

Inflammation	TNFR1/IFN-*γ*/TLR9/IL-1/IL-4/IL-6/IL-10/IL-13	LPS-induced inflammation Lymphocyte transformation test (LTT) Cytokine production Cytokine knockouts
	CD69	Flow cytometry

HLA	MHC I/MHC II	Flow cytometry

Cytochrome P450 (CYP450), uridine 5′-diphosphate (UDP) glycosyltransferase (UGT), glutathione S-transferase (GST), N-acetyl transferase (NAT), sulfotransferase (SULT), glutathione peroxidase (GPx), superoxide dismutase (SOD), 1-chloro-2,4-dinitrobenzene (CDNB), p-nitrobenzyl chloride (NBC), 1,2-chloro-4-nitrobenzene (DCNB), 2-epoxy-3-(p-nitrophenoxy)propane (EPNP), dichloromethane (DCM), trans-4-phenyl-3-buten-2-one (PBO), lipopolysaccharide (LPS), tumor necrosis factor R1 (TNFR1), interferon gamma (IFN-*γ*), toll-like receptor (TLR), interleukin (IL), cluster of differentiation (CD), major histocompatibility complex class I (MHC I), and class II (MHC II).
